# Cardiac Movement of Heart Revealed by a Machine Gun Bullet

**Published:** 1919-01

**Authors:** 


### ABSTRACTS

**Cardiac Movements of Heart Revealed by a Machine Gun Bullet Embedded in the Wall.** By Colonel C. J. Bond, Captain E. V. Philipps, R. A. M. C., and W. Jevons. *Journal of the R. A. M. C.*, September, 1918.

The authors report a case of wound in the chest by a machine gun bullet fired from an aeroplane. The man was, when wounded, lying on his back. An X-ray examination of the chest revealed the presence of a bullet at a depth of about 5 cm., apparently embedded in the wall of the left ventricle near the apex, and moving with systole and diastole, and with the respiratory motions of the diaphragm.

All observed movements of the bullet may be summarized as follows:

(1) *The effect of alterations in bodily position on the position of the heart.* The level assumed by the bullet with the body in the erect position is lower by half the depth of the 11th vertebra than the level assumed with the body in the supine position. The effect of forced inspiration causes the apex of the bullet to point downwards in the erect position, while it is practically horizontal in the supine position. The greatest difference in lateral deviation of the bullet shadow is obtained by placing the patient on his right or left side respectively.

(2) *The effect of respiratory movements on the bullet shadow.* During expiration the motion of the bullet has three components: (*a*) a translation upwards; (*b*) a rotation clockwise, in which the excursion of the apex of the bullet is greater than that of the base; (*c*) a lateral motion of translation.

(3) *The intrinsic movement of the bullet during cardiac systole and diastole.* The apex of the bullet and the left border of the heart tend to approximate during systole. The movement of the base is small, and opposite in direction to that of the apex. The diastolic and systolic relationship in the motion of the bullet is roughly the same in all positions of the heart.

The evidence of the case recorded shows that the heart rests upon, and, by its under surface, moulds itself to the convex dome of the diaphragm, at any rate during the uncontracted diastolic phase. It also occupies the angle between the thoracic wall and the rising diaphragm. The chief movements of the heart brought about by changes in bodily position are lateral side to side movements and up and down alterations in level movements. In all bodily positions, the portion of the heart which lies on the diaphragm is raised, but while in erect, sloping supine, and left-sided positions, this elevation is accompanied by a spreading outwards to the left. In the right-sided position the heart falls backwards and to the right on its own base, and when the diaphragm rises in forced expirations the portion of the heart resting on the diaphragm and the apex rises and passes inwards towards the spine.

